# Data for Disaster Planning: Risk Factors for Internal Radiation Exposures after Fukushima

**DOI:** 10.1289/ehp.122-A166

**Published:** 2014-06-01

**Authors:** Lindsey Konkel

**Affiliations:** Lindsey Konkel is a Worcester, MA–based journalist who reports on science, health, and the environment. She is an editor for *Environmental Health News* and *The Daily Climate*.

Factors determining how much radioactive material people take into their bodies after a major nuclear disaster have not been well defined. In this issue of *EHP* researchers from the University of Tokyo identify risk factors for contamination among residents near the Fukushima Dai-ichi nuclear power plant accident.^1^ They conclude that with the right precautions people might be able to live continuously in radioactively contaminated areas with limited risk of internal exposure.

Studies of the 1986 Chernobyl accident suggested that internal radionuclide exposures from that disaster were due mainly to the ingestion of contaminated food products^2^ and inhalation of radioactive particles.^3^ Risks of dietary exposures may persist long after a nuclear accident, depending on the half-life of radioisotopes, whereas the risk of inhaling radioactive particles from the air is expected to decline more rapidly over time, according to the authors of the current study.

**Figure:**
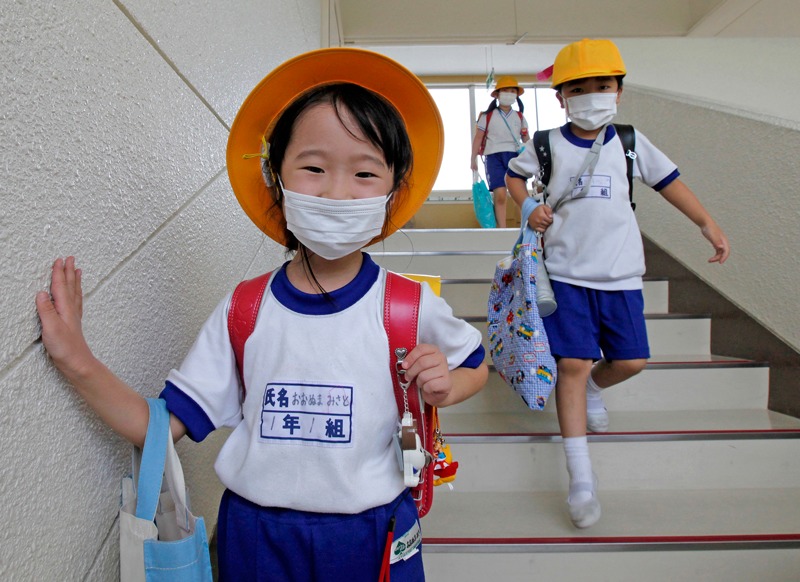
Students at a Minamisoma elementary school six months after an earthquake and tsunami devastated the Fukushima Dai-ichi nuclear power plant. Proportionally fewer Minamisoma children had detectable internal contamination than adults, possibly due to differences in metabolic rates or risk behaviors—a trend also observed following the Chernobyl disaster. © Reuters/Kim Kyung-Hoon

The authors used data for 8,281 residents of Minamisoma City aged 6 years and older. The city is located several miles north of the Fukushima Dai-ichi plant, and residents were told to evacuate in the days after the 11 March 2011 disaster. The evacuation order was lifted in April 2012.^4^ However, 77% of the study participants reported they never left the city.

Participants were members of the Voluntary Internal Radiation Exposure Screening program, which has offered free long-term health monitoring to all Minamisoma residents since the disaster. For a six-month period from October 2011 through March 2012, the researchers collected readings of participants’ levels of cesium-134 and -137 using a whole-body counter. They also administered questionnaires on dietary, occupational, and lifestyle habits.

The researchers found that 40% of adults in the study and 9% of children had detectable levels of internal contamination at the time of screening. Levels in study participants ranged from 2.3 to 196.5 Bq/kg body weight over the six-month study, though the median was on the low end, at 11.3 Bq/kg. Levels over 50 Bq/kg were rare, the authors note, and they declined over time—by March 2012, less than 2% of study participants had any detectable level of internal contamination.

The researchers found that people who spent more time outdoors and reported consuming more local foods and tap water were significantly more likely to have higher levels of contamination. The purchase of non-Fukushima-produced rice was significantly associated with lower contamination. However, the authors have also reported elsewhere that because contamination of local foods and tap water decreases over time, the association between internal contamination and consumption gradually disappears.^5^^,^^6^

In Fukushima, as with other nuclear accidents, cesium was one of the radioactive particles released in the greatest amounts.^7^ The investigators found no evidence of acute health problems stemming from cesium exposure among residents, although it’s difficult to predict long-term health effects based on the information they collected.

“At the levels they’re looking at, iodine is probably the more important exposure,” says Bruce Napier, an environmental health physicist at Pacific Northwest National Laboratory in Washington, who was not involved with the study. Radioiodine exposure has been associated with adverse health effects including thyroid cancer,^1^^,^^8^ benign thyroid follicular adenomas,^9^ and functional thyroid disorders.^7^^,^^10^

However, the researchers were unable to assess exposures to radioiodine, which has a half-life of just 8 days. They also note that the self-selected study population may not have been representative of Minamisoma City residents as a whole; for instance, health-conscious residents may have been more likely both to protect themselves against exposure and to seek screening.

Despite these limitations, “the study is very useful in that it presents an underlying plan for the future and shows what aspects of response may be most beneficial to public health,” says Lydia Zablotska, an epidemiologist at the University of California, San Francisco, who also was not involved with the study.

The researchers suggest that strict food and water regulations and campaigns to increase awareness of food origin implemented soon after the Fukushima disaster may have helped decrease contamination risk. In the past, nuclear disaster management plans have predominantly involved evacuation and relocation, they note, and studies from Chernobyl suggest that weak food and water regulations were the major culprits in prolonged internal exposure and increased cancer rates in the aftermath of that disaster.^8^^,^^11^

The low levels of internal contamination of people in Minamisoma suggest it may be possible to control exposure levels if good food restriction systems are adhered to, according to lead author Amina Sugimoto, who is currently pursuing a doctoral degree at the London School of Hygiene and Tropical Medicine. “Mass evacuations such as happened in Chernobyl and Fukushima may not be the best approach apart from areas with extreme levels of airborne radiation,” Sugimoto says.
